# Stability of Lysozyme in Aqueous Extremolyte Solutions during Heat Shock and Accelerated Thermal Conditions

**DOI:** 10.1371/journal.pone.0086244

**Published:** 2014-01-23

**Authors:** Christina Avanti, Vinay Saluja, Erwin L. P. van Streun, Henderik W. Frijlink, Wouter L. J. Hinrichs

**Affiliations:** 1 Department of Pharmaceutics, Faculty of Pharmacy, University of Surabaya (Ubaya), Surabaya, Indonesia; 2 Department of Pharmaceutical Technology and Biopharmacy, University of Groningen, Groningen, The Netherlands; 3 Pharmaceutical Sciences and Clinical Supply (PSCS), Development Center Oss, MSD, Oss, The Netherlands; Russian Academy of Sciences, Institute for Biological Instrumentation, Russian Federation

## Abstract

The purpose of this study was to investigate the stability of lysozyme in aqueous solutions in the presence of various extremolytes (betaine, hydroxyectoine, trehalose, ectoine, and firoin) under different stress conditions. The stability of lysozyme was determined by Nile red Fluorescence Spectroscopy and a bioactivity assay. During heat shock (10 min at 70°C), betaine, trehalose, ectoin and firoin protected lysozyme against inactivation while hydroxyectoine, did not have a significant effect. During accelerated thermal conditions (4 weeks at 55°C), firoin also acted as a stabilizer. In contrast, betaine, hydroxyectoine, trehalose and ectoine destabilized lysozyme under this condition. These findings surprisingly indicate that some extremolytes can stabilize a protein under certain stress conditions but destabilize the same protein under other stress conditions. Therefore it is suggested that for the screening extremolytes to be used for protein stabilization, an appropriate storage conditions should also be taken into account.

## Introduction

Protein instability is one of the main issues in the administration of therapeutic protein based medicines, in particular in aqueous formulations. A number of experimental studies have been done to overcome protein instability. One of the most promising results is the discovery of extremolytes, small organic osmolytes found in extremophiles [1]–[3].

**Figure 1:**
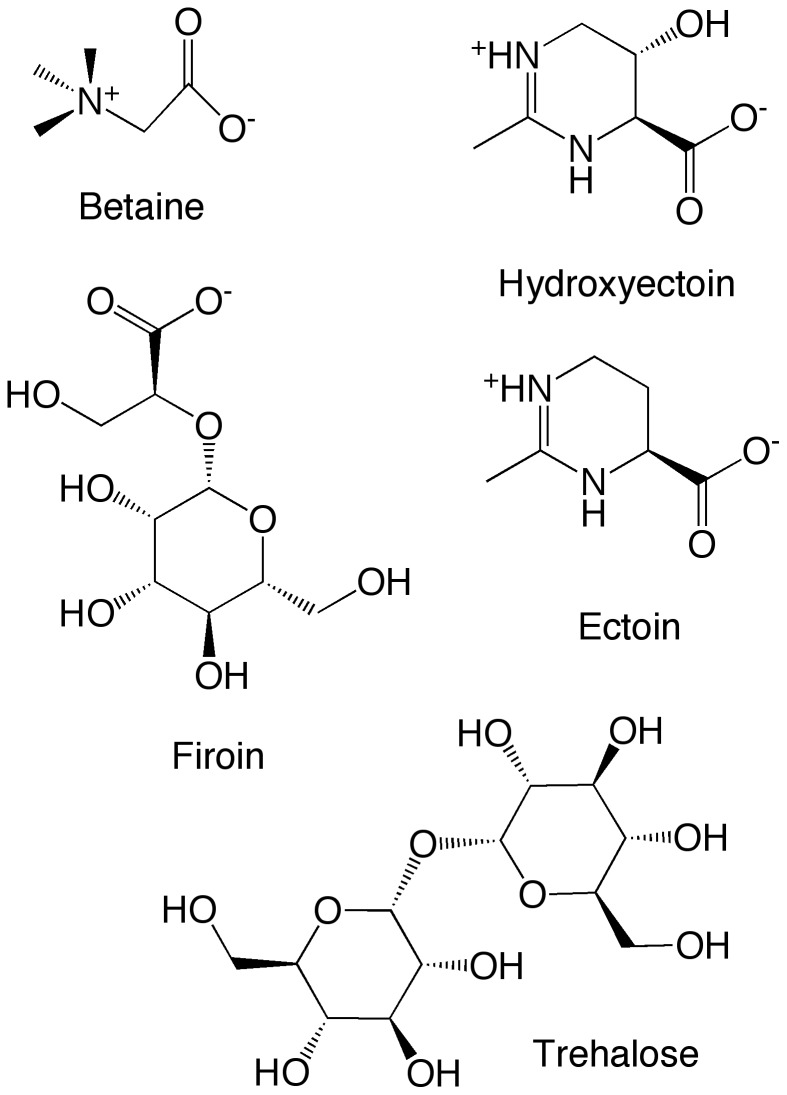
Molecular structure of the extremolytes betaine, hydroxyectoine, trehalose, ectoine, and mannosylglycerate (firoin).

Extremophiles are microorganisms which are capable of surviving under extreme conditions, such as high or low temperatures, extreme pressure and high salt concentrations. Extremolytes are accumulated in response to these extreme conditions and protect the biomacromolecules such as proteins against denaturation in vivo [1], [4]. Various molecules that have been identified as extremolytes i.e. the polyol derivatives ectoine, hydroxyectoine, betaine [5], various amino acids [6], carbohydrates such as trehalose [7], and the mannose derivative firoin [8], [9]. Figure 1 shows the molecular structures of some of these extremolytes.

The fact that extremolytes can stabilize biomacromolecules in microorganisms leads to the hypothesis that these molecules could also stabilize proteins in vitro. Índeed it has been found that a number of extremolytes were also able to stabilize proteins in aqueous solutions [10], [11].

**Figure 2:**
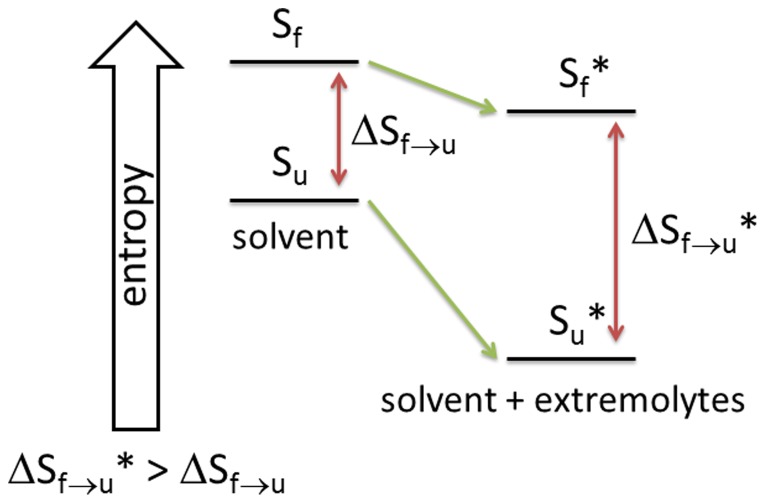
Proposed mechanism of stabilization of extremolytes, adapted from Arakawa et al. [12]. In the unfolded state (S_u_) proteins undergo the greatest loss of its conformational entropy, which led to the overall shift in equilibrium towards the native state. The stabilization occurs when the entropy difference between two states increases, so that the folded protein (S_f_) has fewer tendencies to unfold [11], [12].

Extremolytes stabilize proteins because they are preferentially excluded from the surface of the protein molecules [12], [13]. Exclusion occurs as a result of the repulsive interactions between the extremolytes and the backbone of the protein molecules [14] by which water molecules are accumulated near the surface of the protein molecules. As a result of preferential exclusion of the extremolytes from the surface of the protein molecules, proteins turn into a more compact tertiary structure with a reduced surface area. According to this mechanism, in the presence of extremolytes the native state of proteins is in the lower free energy state than in the unfolded state. Proteins lose their conformational entropy with the greater entropic loss in the unfolded state (S_u_), leading to an overall shift in equilibrium towards the native state [15]. The entropy difference between the two states increases, thus the folded protein (S_f_) is less likely to unfold as ilustrated in Figure 2 [16]. Borges et al. have evaluated extremolytes for their stabilizing capabilities on lactate dehydrogenase [9]. It was found that firoin can prevent lactate dehydrogenase aggregation and thus inactivation during heat stress. It was also found that the melting temperature of lactate dehydrogenase increased by 4.5°C in the presence of firoin at a concentration of 0.5 M, whereas trehalose caused an increase of the melting temperature of only 2.2°C at the same concentration [9]. Faria et al. investigated the effect of extremolytes on the thermal unfolding of RNase A and found that at a concentration of 0.5 M firoin was an efficient thermostabiliser for this protein and induced an increase of the melting temperature by 3.2°C and 4.1°C at a pH of 4.5 and 7.0, respectively. The stabilizing capacities of firoin were attributed to a decrease in the unfolding entropy [8].

Previous studies clearly showed that extremolytes can be used as pharmaceutical excipients to improve the stability of protein based medicines in aqueous formulations. The aim of this study was to investigate whether known extremolytes such as betaine, hydroxyectoine, trehalose, ectoine, and firoin are able to stabilize the model protein lysozyme in aqueous solutions during two different stress conditions i.e. heat shock (incubation at 70°C for 10 minutes) and accelerated thermal conditions (incubation at 55°C for 4 weeks). Lysozyme was selected as a model protein as it has been extensively studied, it can be obtained in high purity, and its enzymatic activity can be determined relatively easily.

## Materials and Methods

### Materials

The following materials were used in this study: Hen egg-white lysozyme (Sigma-Aldrich, Steinheim, Germany), ultrapure trehalose (Cargill, Krefeld, Germany), ultrapure betaine (Fluka Biochemika, Buchs, Switzerland), firoin (Biotop, Berlin-Brandenburg, Germany), ultrapure ectoine and hydroxyectoine (Biomol, Hamburg, Germany), citric acid (Riedel-de Haen, Seelze, Germany), sodium hydroxide, dimethylsulfoxide (DMSO), Nile red and *M. lysodeickticus* (Sigma-Aldrich, Steinheim, Germany), phosphate buffered saline (PBS buffer 66 mM phosphate, pH 6.2) (Fluka Analytical, Steinheim, Germany), SYPRO orange protein gel stain, (Invitrogen, Eugene, Oregon, USA),

### The effect of extremolytes on the stability of lysozyme

#### Heat shock stability study

Lysozyme solutions at a concentration of 100 µg/ml were prepared in 10 mM citrate buffer (pH 5.0) with and without extremolytes at a concentration of 0.5 M [1], [8]. Lysozyme solutions were incubated at 70°C for 10 minutes. The effect of extremolytes on the stability of lysozyme was determined by Nile red fluorescence spectroscopy and by measuring its bioactivity.

#### Thermal degradation studies

Lysozyme solutions at a concentration of 100 µg/ml were prepared in 10 mM citrate buffer (pH 5.0) with and without 0.5 M extremolytes. Lysozyme solutions were incubated at 55°C. The effect of extremolytes on the stability of lysozyme was determined by measuring its bioactivity. Bioassays were performed every week for 4 weeks.

#### Nile red Fluorescence Spectroscopy

Fluorescence studies were performed on a SLM-Aminco AB2 Spectrofluorometer at the temperature of 25°C using 5 mm cubical quartz cuvette (Hellma GmbH). Prior to measurement, 5 µl of 20 µg/ml Nile red solution in DMSO was added to a 1 ml of 100 µg/ml lysozyme solution, to obtain a Nile red : lysozyme weight ratio of 1∶1000. An excitation wavelength of 550 nm was used, with a band pass of 4.0 nm for the excitation monochromator and an emission wavelength of 610 nm with a band pass of 4.0 nm for the emission monochromator. Data were recorded at 1 nm intervals over the range of 560–700 nm with a scanning speed of 100 nm/min. Spectra were corrected for background signal caused by buffer and extremolytes [17].

#### Bioassay

The bioactivity of lysozyme was determined by measuring the rate of lysis of *Micrococcus lysodeikticus*by using a method as described by Shugar [18] with some modifications. Briefly, 1.3 ml of a 200 µg/ml of *Micrococcus lysodeikticus* suspension in sterile PBS buffer (66 mM phosphate, pH 6.2) was mixed with 10 µl of the lysozyme solutions in plastic disposable cuvettes. Immediately after mixing, the cuvette was placed in a UV/VIS spectrophotometer and the absorbance was measured at a wavelength of 450 nm. Subsequently, the change of the absorbance was recorded over time. The activity of the stresses lysozyme samples were compared with that of unstressed lysozyme. Statistical analyses were performed using Student's *t* test with *p*<0.05 as the minimal level of significance. The results are presented as mean ± standard error mean unless indicated otherwise.

### Unfolding temperature (*T*_m_)

The unfolding temperature (*T*_m_) of lysozyme in solutions was analyzed by a thermal shift assay using a real-time PCR machine [19]. Sypro Orange has been used as environmetally sensitive fluorescence dye that quenched in an aqueous solution. When the protein unfolded, the hydrophobic core region is exposed to the dye. The fluorescence then monitored as a function of the temperature. The midpoint of the protein unfolding transition is defined as *T*_m_. Solutions of 17.5 µl of 1.0 mg/ml lysozyme with or without extremolytes (1 M) and 7.5 µl of 300 fold diluted SYPRO Orange solution as molecular probes were added to the wells of a 96-well thin-wall PCR plate (Bio-Rad). The plates were sealed with optical-quality sealing tape (Bio-Rad Laboratories BV, Veenendaal, The Netherlands), inserted into a real-time PCR machine (iCycler, Bio-Rad Laboratories BV, Veenendaal, The Netherlands) and heated from 20 to 90°C with a 0.2°C increase per 20 s. The fluorescence changes of the SYPRO Orange probe in the wells of the plate were monitored simultaneously with a fluorescence detector (MyIQ single-colour RT-PCR detection system, Bio-Rad) at excitation and emission wavelengths of 490 and 575 nm, respectively. The midpoint of the transition was taken as the *T*_m_.

### Isothermal Titration Calorimetry (ITC)

Microcalorimetric titrations of extremolytes to lysozyme in citrate buffer pH 5.0 were performed by using a MicroCal ITC_200_ microcalorimeter (Northampton, MA 01060 USA). A solution of 20 mM of selected extremolyte in 10 mM citrate buffer pH 5.0 was placed in the syringe, while 300 µl of 1 mM lysozyme in 10 mM of citrate buffer, pH 5.0 was placed in the sample cell. The reference cell contained 300 µl of citrate buffer. Experiments were performed at 10, 25, and 55°C. The initial delay time was 60 s. The reference power and the filter were set to 5 µcal/s and 2 s respectively. A typical titration experiment consisted of 20 injections of 2 µl extremolytes solutions with duration of 4 s and the time interval between two consecutive injections was set to 180 s. During the experiments, the sample solution was continuously stirred at 1000 rpm. The effective heat of the protein-extremolytes interaction upon each titration step was corrected for dilution and mixing effects, as measured by titrating the extremolytes solution into the buffer and by titrating the buffer into the protein solution. The heats of bimolecular interactions were obtained by integrating the peak following each injection. All measurements were performed in triplicate. ITC data were analyzed by using the ITC non-linear curve fitting functions for one or two binding sites from MicroCal Origin 7.0 software (MicroCal Software, Inc.) [20].

## Results and Discussion

### The effect of extremolytes on the stability of proteins

In order to investigate the effect of the different extremolytes on the stability of lysozyme, Nile Red Fluorescence Spectroscopy and a bioactivity assay were used. In all studies an extremolyte concentration of 0.5 M was used following previous studies [8], [21]. pH 5.0 were used based on the study of the melting temperature of RNase A in various pH that found that above pH 5 the melting temperature of RNase A remains approximately constant either in the absence or presence of extremolytes [8] and also for lysozyme that exhibited maximum thermal stability at pH 5.0 [22]. In the literature most lysozyme stability studies were performed in citrate [22]–[24] and that indeed we found in preliminary studies citrate gave the most reproducible results.

#### Stability of lysozyme as measured by Nile Red Fluorescence Spectroscopy

**Figure 3:**
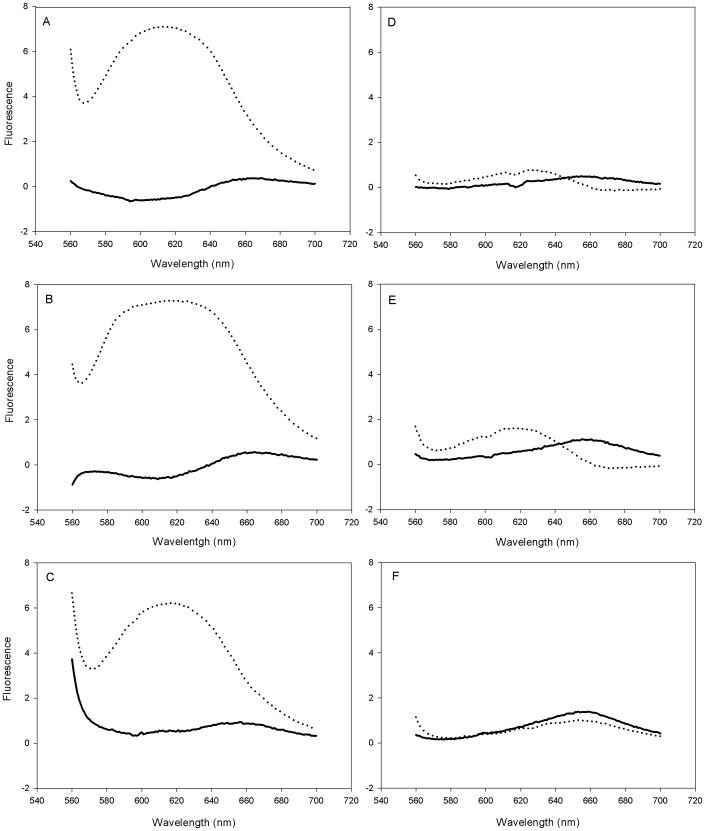
The effect of extremolytes on the Nile Red fluorescence of the lysozyme-nile red complex after stressing lysozyme at 70°C for 10 minutes (heat shock) in citrate buffer pH 5.0. Fluorescence spectra recorded on unstressed (solid line) and stressed (dotted line) lysozyme A: without extremolytes, and with extremolyte B: betaine, C: hydroxyectoine, D: trehalose, E: ectoine, and F: firoin.

Nile red fluorescence can be employed to probe changes in protein conformations that are related to the formation of hydrophobic surfaces, such as during aggregation or protein unfolding because of its sensitivity to the polarity of its environment [25], [26]. The peak maximum of unstressed lysozyme is about 290 nm and an intensity of about 0.5. The effect of a heat shock on lysozyme without extremolytes in citrate buffer solution pH 5.0 is shown in Figure 3A. A huge increase in the fluorescence intensity of Nile red was observed when lysozyme without extremolytes was stressed at 70°C for 10 minutes indicating substantial denaturation of lysozyme. Apparently, heating lysozyme without extremolytes for 10 minutes at 70°C caused collapse of its secondary and/or tertiary structure. Stressed lysozyme solutions in the presence of betaine (Figure 3B) or hydroxyectoine (Figure 3C) showed similar Nile red fluorescence spectra. The peaks did not only show a large shift from about 290 nm to about 620 nm (a so-called blue shift) but also a strong increase of their intensity from about 0.5 to about 5–7. However, when trehalose was added, we observed a slight difference in the Nile red fluorescence spectra of the lysozyme sample before and after heat shock. Figure 3D shows that there was a minor shift in the maximum peak after stress, however, the huge increase in the fluorescence intensities of Nile red as found for lysozyme formulations without extremolytes or in the presence betaine or hydroxyectoine was not observed. This indicates that trehalose was able to inhibit substantial denaturation of lysozyme. The stabilization effect of trehalose might be due to the fact that trehalose has a propensity to depress the formation of aggregates and chemical reactions of lysozyme by inducing α-helical structures and some tertiary structures [2]. Also the Nile red fluorescence spectrum of the lysozyme solution formulated with ectoine showed a minor shift in the maximum peak after stress (Figure 3E). Furthermore, also significantly higher fluorescence intensity was observed in the stressed lysozyme samples containing ectoine than those containing trehalose. In Figure 3D and 3E only the blue shift but no substantial increase in the intensity of the peaks can be observed. Thus in these cases there was a change in structure of lysozyme, however, this change was much smaller than found for formulations without extremolytes or in the presence betaine or hydroxyectoine (Figure 3A–C). Therefore, these measurements indicate that ectoine and trehalose protected lysozyme against denaturation much better than when no extromolyte was used or in the presence betaine or hydroxyectoineWhen firoin was added to the lysozyme solution, there was neither a blue shift nor a change in intensity of the peak (Figure 3F). As the Nile red fluorescence spectra of unstressed and stressed lysozyme solution were fully identical, we can conclude that firoin completely inhibited the denaturation of lysozyme during heat shock.

#### Stability of lysozyme determined by using Bioassay

**Figure 4:**
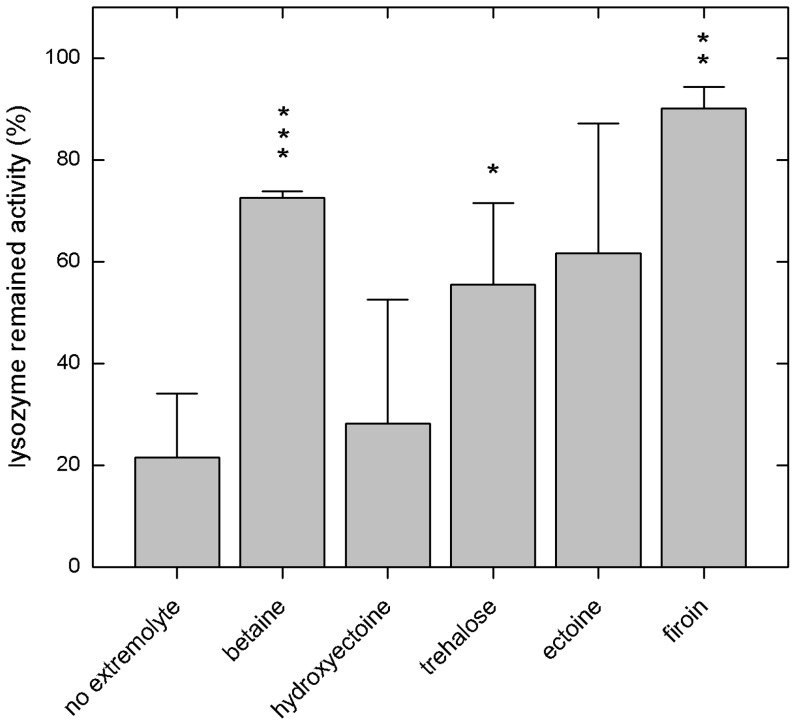
The effect of lysozyme on *M. Lysodeickticus* (bioactivity) after stressed at 70°C for 10 minutes (heat shock) and the effect of extremolytes on the bioactivity of lysozyme. * is the level of significance (*p*<0.05).

In order to further evaluate the stabilizing effects of extremolytes during a heat shock at a temperature of 70°C for 10 minutes and during storage at a temperature of 55°C for 4 weeks, the biological activity of lysozyme was determined. After heat shock the ability of lysozyme to inactivate the bacterium *M. Lysodeickticus* decreased dramatically. Figure 4 shows that lysozyme only maintained about 20–40% of its original activity. When betaine, trehalose, and ectoine were added, however, lysozyme maintained about 70% of its original activity. There was no significant difference observed between the stabilizing effects of hydroxyectoine, trehalose, and ectoine but it is difficult to draw a conclusion from these data since the level of significance was low. It seems, however, that ectoine stabilized lysozyme better than trehalose and hydroxyectoine, also hydroxyectoine was not significantly different from control.

**Figure 5:**
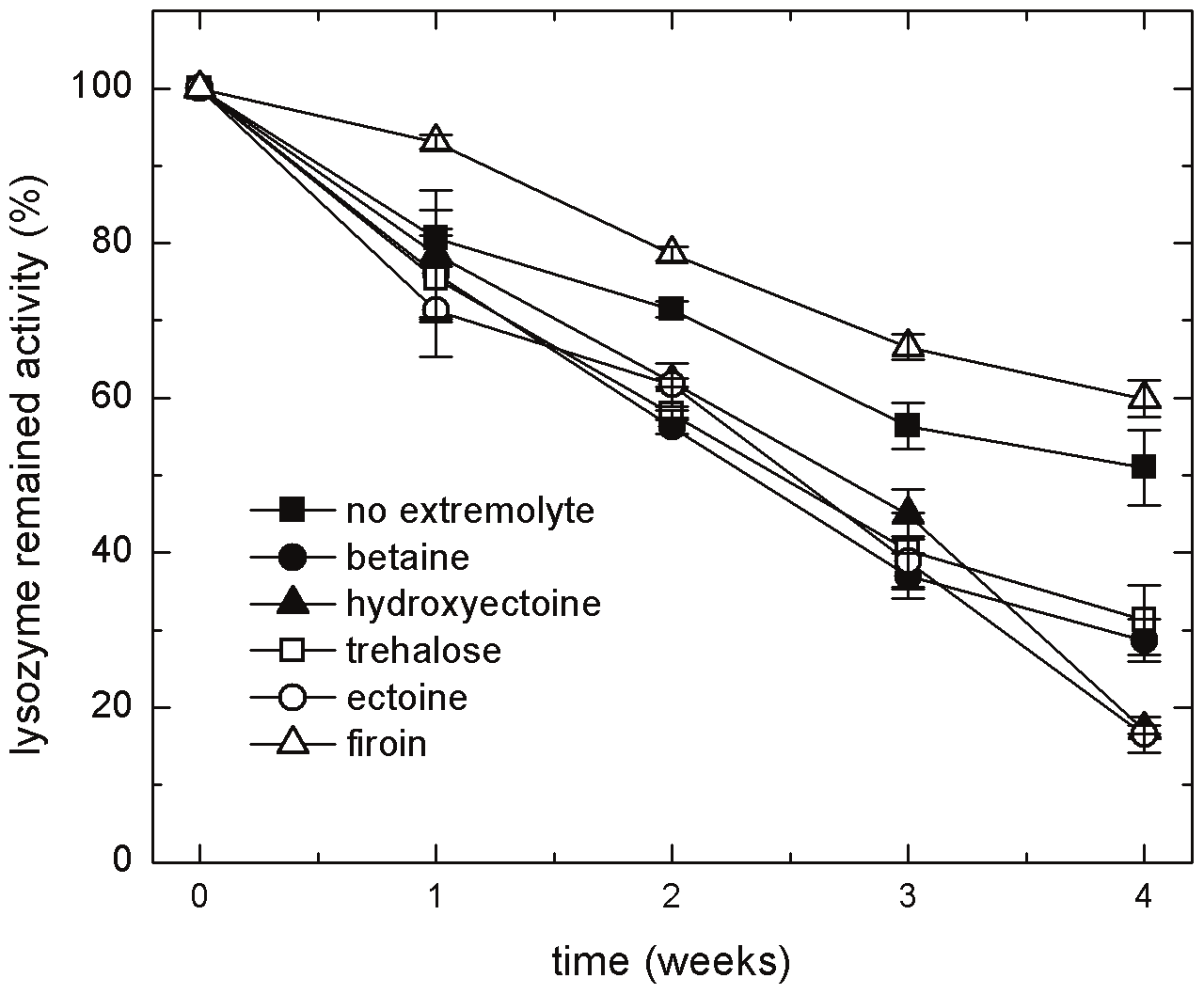
The effect of extremolytes on the bioactivity of lysozyme during 4 weeks of storage at 55°C (accelerated thermal condition).

The bioactivity assay was also used to monitor the effects of extremolytes on the stability of lysozyme solution during incubation for 4 weeks at 55°C (accelerated thermal condition). Figure 5 shows that the addition of the extremolytes betaine, hydroxyectoine, trehalose and ectoine destabilized lysozyme. These results are in contrast to the heat shock experiments where these extremolytes lead to minor or substantial stabilizing effects. On the other hand, the bioactivity assay indicated that firoin stabilized lysozyme during storage at a 55°C for 4 weeks, which is completely in line with the results of the heat shock experiments.

### The effect of extremolytes on protein unfolding temperature (*T*_m_)

**Figure 6:**
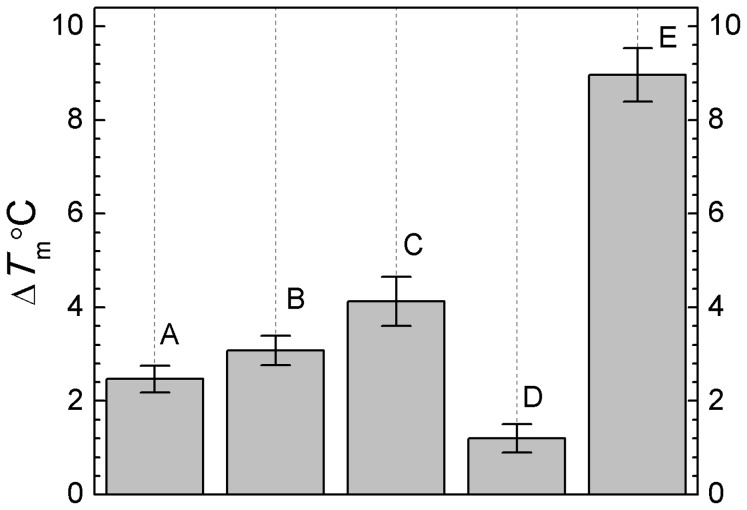
The effect of extremolytes on the unfolding temperature (*T*_m_) of lysozyme. Unfolding temperatures was measured as transition midpoint analyzed by thermal shift assay using RT-PCR machine of lysozyme on the concentration of 1.0 mg/ml with A: betaine, B: hydroxyectoine, C: trehalose, D: ectoine, and E: firoin.

As the temperature increases, lysozyme undergoes thermal unfolding. The *T*_m_ for lysozyme was found to be 82°C. Both experimental temperatures (55 and 70°C) are much lower than the lysozyme *T*_m_ in water (82°C). According to the two-state unfolding model, the enzyme should maintain more than 50% of folded state in these conditions. However, at 70°C for short period we had full unfolding which we saw with fluorescence/bioassay as well as with 55°C for 4 weeks. The addition of firoin resulted in a much higher increase in the *T*_m_, i.e. 9°C (Figure 6), while the other extremolytes which generated an increase of the *T*_m_ of maximally 4°C only. These results may explain the improved stability of lysozyme in the presence of firoin. The firoin effect may be due to the increase of the melting temperature of lysozyme by 9°C. It may be that a rise in *T*_m_ of 4°C is not enough to protect lysozyme for a longer period of time at 55°C, but that a rise of 9°C is enough to stabilize lysozyme for at least a week at 55°C. Such findings have also been observed in studies of thermal denaturation of lysozyme in aqueous solution of polyvalent alcohol and sugar, which can stabilize the lysozyme native conformation [27]–[29]. The increase in *T*_m_ about 18.5°C at the presence of 50% (w/w) of sorbitol stabilizes lysozyme in aqueous solution [27]. Santoro *et al.* showed that up to 8.2 molar of extremolytes, including betaine, were able to increase the *T*_m_ of lysozyme to about 23°C [11]. It is possible that higher extremolytes concentrations are required to improve the stability of lysozyme during storage at a high temperature.

### Interaction of proteins with extremolytes

**Figure 7:**
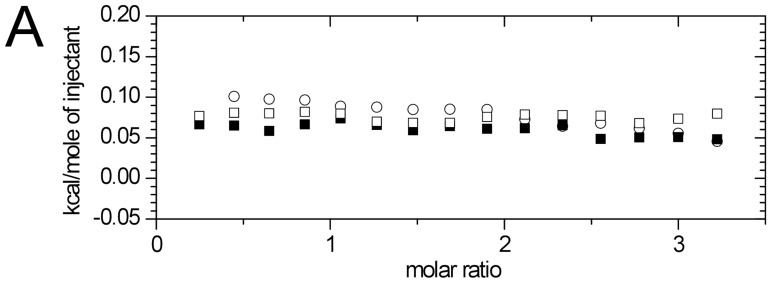
The effect of firoin on the heat capacity of lysozyme determined by ITC. Firoin was titrated into lysozyme solution at the temperatures of 10°C (▪), 25°C (□), or 55°C (○).

The interaction of extremolytes with proteins was analyzed using *Isothermal Titration Calorimetry* at different temperatures for the most stable formulations. Figure 7 shows the similarity in the magnitude of the reaction and dilution enthalpy for the titration of lysozyme by firoin at the temperatures of 10, 25, or 55°C. The results indicate that in aqueous solution firoin did not show any significant interaction with the protein. This is in line with the preferential hydration theory that the repulsion between the amide backbone of the protein and the extremolytes is due to the influence of the extremolytes on the water structure and therefore do not interact directly with the proteins [1].

### Conclusion

This study clearly shows that certain extremolytes (firoin) can act as a stabilizer for lysozyme. Certain extremolytes (betaine) can stabilize a protein (lysozyme) under certain conditions (heat shock) but destabilize the same protein under other stress conditions (accelerated thermal conditions). This implies that for the screening of extremolytes for the stabilization of proteins the envisaged storage conditions should be taken into account. Furthermore, for screening extremolytes for the stabilization of proteins, measuring the *T_m_* of the protein can be useful to predict stabilizing effects but only if the extremolyte induces a substantial change in the *T_m_*.
